# Prediction Model Development and Validation of 12-Year Incident Edentulism of Older Adults in the United States

**DOI:** 10.1177/23800844221112062

**Published:** 2022-08-09

**Authors:** J.S. Preisser, K. Moss, T.L. Finlayson, J.A. Jones, J.A. Weintraub

**Affiliations:** 1 Biostatistics, University of North Carolina Gillings School of Global Public Health, Chapel Hill, NC, USA; 2 Division of Comprehensive Oral Health University of North Carolina at Chapel Hill, Adams School of Dentistry, Chapel Hill, NC, USA; 3 Health Management and Policy, San Diego State University School of Public Health, San Diego, CA, USA; 4 University of Detroit Mercy, Detroit, MI, USA; 5 Division of Pediatric and Public Health, University of North Carolina at Chapel Hill, Adams School of Dentistry, Chapel Hill, NC, USA

**Keywords:** tooth loss, oral health, aged, geriatrics, incidence, epidemiology

## Abstract

**Introduction:**

Edentulism affects health and quality of life.

**Objectives:**

Identify factors that predict older adults becoming edentulous over 12 y in the US Health and Retirement Study (HRS) by developing and validating a prediction model.

**Methods:**

The HRS includes data on a representative sample of US adults aged >50 y. Selection criteria included participants in 2006 and 2018 who answered, “Have you lost all of your upper and lower natural permanent teeth?” Persons who answered “no” in 2006 and “yes” in 2018 experienced incident edentulism. Excluding 2006 edentulous, the data set (*n* = 4,288) was split into selection (70%, *n* = 3,002) and test data (30%, *n* = 1,286), and Monte Carlo cross-validation was applied to 500 random partitions of the selection data into training (*n* = 1,716) and validation (*n* = 1,286) data sets. Fitted logistic models from the training data sets were applied to the validation data sets to obtain area under the curve (AUC) for 32 candidate models. Six variables were included in all models (age, race/ethnicity, gender, education, smoking, last dental visit) while all combinations of 5 variables (income, alcohol use, self-rated health, loneliness, cognitive status) were considered for inclusion. The best parsimonious model based on highest mean AUC was fitted to the selection data set to obtain a final prediction equation. It was applied to the test data to estimate AUC and 95% confidence interval using 1,000 bootstrap samples.

**Results:**

From 2006 to 2018, 9.7% of older adults became edentulous. The 2006 mean (SD) age was 66.7 (8.7) for newly edentulous and 66.3 (8.4) for dentate (*P* = 0.31). The baseline 6-variable model mean AUC was 0.740. The 7-variable model with cognition had AUC = 0.749 and test data AUC = 0.748 (95% confidence interval, 0.715–0.781), modestly improving prediction. Negligible improvement was gained from adding more variables.

**Conclusion:**

Cognition information improved the 12-y prediction of becoming edentulous beyond the modifiable risk factors of smoking and dental care use, as well as nonmodifiable demographic factors.

**Knowledge Transfer Statement:**

This prediction modeling and validation study identifies cognition as well as modifiable (dental care use, smoking) and nonmodifiable factors (race, ethnicity, gender, age, education) associated with incident complete tooth loss in the United States. This information is useful for the public, dental care providers, and health policy makers in improving approaches to preventive care, oral and general health, and quality of life for older adults.

## Introduction

While there has been a significant decline in the prevalence and incidence of total tooth loss since 1990, both nationally and around the world (Kassebaum et al. 2014), poor oral health, partial tooth loss, and edentulism remain problems among older adults. Tooth retention is critical for speaking and eating. Tooth loss and edentulism diminish quality of life, health, and functional status. Among older adults, impaired chewing ability is associated with tooth loss (Warren et al. 2002; Ervin and Dye 2012; Tan et al. 2016; Cho and Kim 2019).

Oral health–related quality of life (OHQOL) has been called a “neglected aspect” of overall quality of life (Rouxel et al. 2018). A systematic review found consistent evidence that number of teeth affects OHQOL (Steele et al. 2004). A meta-analysis reported a similar relationship; furthermore, the distribution of tooth loss can mean a lack of functional dentition (Tan et al. 2016) and low OHQOL (Gerritsen et al. 2010). Zhang et al. (2018), using 2008 Health and Retirement Study (HRS) data, found that those with poor oral health exhibited a greater subsequent decline (2008–2014) in functional status.

Complete tooth loss (edentulism) is also associated with a host of noncommunicable diseases, including malnutrition, obesity, cardiovascular diseases, diabetes, respiratory diseases, and cancers (Emami et al. 2013; Felton 2016). Many cross-sectional studies exist (National Institutes of Health [NIH] 2021); cross-sectional data collected over time reported in the extensive 2021 National Institute of Dental and Craniofacial Research report, *Oral Health in America: Advances and Challenges* (NIH 2021), support the use of demographic data, age, gender, race/ethnicity, and education in the study of edentulism as well as smoking, poverty, and diabetes. Similarly, edentulism is associated with infrequent dental care use using the US 2017 Medical Expenditure Panel Survey of adults age 50 y and older (Foiles Sifuentes et al. 2020). Dental care is important even with a lack of natural teeth to maintain the fit of dentures and monitor health changes in the mouth (e.g., oral cancer).

Longitudinal oral health data for cohorts of older adults are scarce but provide a temporal sequence for valid disease prediction models (Peres et al. 2020). A systematic review of observational studies and meta-analysis of sociodemographic determinants of edentulism was conducted by Roberto and colleagues (2019). Of the 24 articles included in their review, 21 were cross-sectional studies; 1 of the 3 cohort studies was US based but conducted in 1994. The review’s authors discussed the importance of longitudinal studies to understand causal factors. Piggybacking on extant longitudinal studies represents an important source of knowledge in this respect.

Our previous work examined incident edentulism in the US HRS from 2006 to 2012 (Weintraub et al. 2019). We found that adults who became edentulous after these 6 y were more likely Black/African American compared to White, were less educated, were current smokers, had diabetes, and reported poorer self-rated general health, more functional limitations and disabilities, and fewer dental visits (all *P* < 0.0001), among other factors. More recently, Cooray et al. (2021) used machine learning to examine the importance of socioeconomic factors in predicting tooth loss in a Japanese longitudinal study, with the intent on better understanding predictors in order to better develop prevention strategies. With similar intent, the purpose of this study was to use data from a longitudinal cohort study to identify factors that predict older adults becoming edentulous over a 12-y period. Our focus included modifiable factors in the nationally representative HRS data (2006–2018), which could provide the basis for future preventive strategies. We used Monte Carlo cross-validation based on randomly split samples to develop and validate a multivariable prognostic prediction model.

## Methods

### Design

The investigators conducted a longitudinal cohort secondary data analysis and did not have any contact with study participants. Multiple waves of deidentified HRS data are available publicly online. This project was reviewed by the University of North Carolina at Chapel Hill Office of Human Research Ethics and was determined not to be human subjects research. We used the T**ransparent Reporting of a multivariable prediction model for Individual Prognosis Or Diagnosis (**TRIPOD) guidelines for prediction modeling studies (Collins et al. 2015).

### Data Source

The HRS is sponsored by the National Institute on Aging (grant number U01AG009740) and is conducted by the University of Michigan (https://hrs.isr.umich.edu/data-products). The HRS is an ongoing, longitudinal study that began in 1992. It is a nationally representative sample of US adults over age 50 y. Approximately 20,000 people participate over time; additional cohorts are periodically recruited. Analyses for this study used biennial Core data from 2006 to 2018 that were collected using face-to-face and telephone interviews.

### Eligibility Criteria, Sample Size, and Outcome Measure

**Figure 1.**
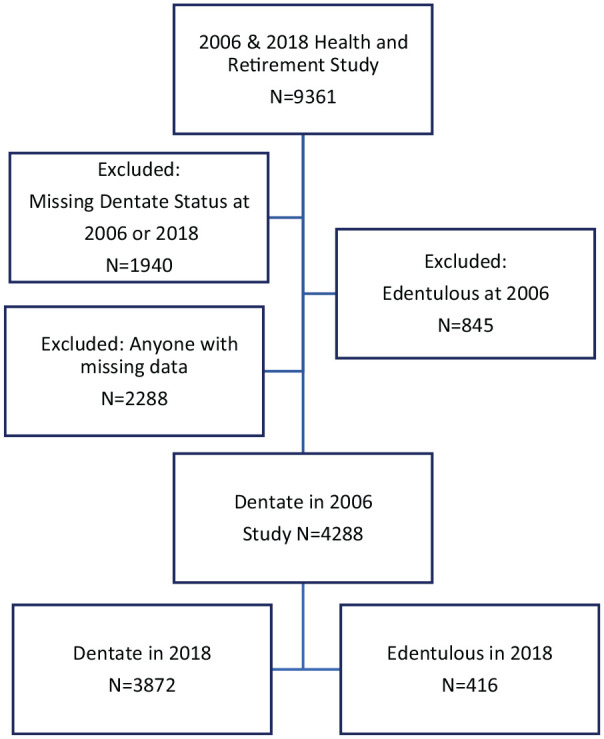
Study participants from the Health and Retirement Survey.

A question about complete tooth loss was first asked in 2006 and again in 2012 and 2018. Inclusion criteria were adults >50 y in 2006 who answered the tooth loss question in both 2006 and 2018. Respondents who answered “yes” to the question, “Have you lost all of your upper and lower natural permanent teeth?” were considered edentulous and dentate if they responded “no.” Because our goal was to predict incident edentulism, adults already edentulous in 2006 were excluded from analysis (*n* = 845). Persons with incomplete data for our outcome or explanatory variables were also excluded (*n* = 2,288). Our primary outcome was 12-y edentulism incidence. Participants who answered the tooth loss question with “no” in 2006 and “yes” in 2018 became edentulous during this 12-y period, and those who remained dentate comprised the final analytical sample (*n* = 4,288; Fig. 1).

### Explanatory Variables

We selected an initial set of 6 established explanatory variables from the 2006 survey based on their known association with tooth loss and edentulism in the literature, including the traditional demographic factors (age, gender, race/ethnicity, and education) and modifiable factors of smoking and use of dental care. A recent dental visit was based on the response to the question, “In the last 2 years, have you seen a dentist for dental care, including dentures?”

### Candidate Predictors

Five candidate predictor variables from the 2006 survey were considered potential predictors of 12-y edentulism: cognitive status, alcohol use, self-rated health, loneliness, and income. The categorization of predictor variables and their frequency distributions are shown in Table 1. Categories were defined considering univariate 2006 data only.

**Table 1.** Frequency Distribution of 2006 Characteristics of Participants by Dentate Status in 2018.

Characteristic in 2006	Dentate in 2018	Became Edentulous between 2006 and 2018	*P* Value
Total	3,872 (90.3)	416 (9.7)	
Race/ethnicity			
Caucasian	2,964 (92.7)	234 (7.3)	
African American	454 (83.0)	93 (17.0)	
Hispanic	363 (82.3)	78 (17.7)	
Other	91 (89.1)	11 (10.8)	<0.0001
Gender			
Female	2,265 (90.9)	226 (9.1)	
Male	1,607 (89.4)	190 (10.6)	0.10
Age, mean (SD), y (in 2006)	66.3 (8.4)	66.7 (8.7)	0.31
Education/degree			
No high school degree	491 (79.8)	124 (20.2)	
< College	2,222 (89.8)	252 (10.2)	
College +	1,159 (96.7)	40 (3.3)	<0.0001
Smoking			
Current	299 (75.1)	99 (24.9)	
Former	1,616 (90.0)	180 (10.0)	
Never	1,957 (93.5)	137 (6.5)	<0.0001
Last dentist visit			
Within 2 y	3,096 (93.3)	223 (6.7)	
>2 y	776 (80.1)	193 (19.9)	<0.0001
Ever drink alcohol			
Yes	2,302 (92.5)	188 (7.6)	
No	1,570 (87.3)	228 (12.7)	<0.0001
Total cognition score			
<23	1,188 (83.4)	236 (16.6)	
23+	2,684 (93.7)	180 (6.3)	<0.0001
Self-rated health			
Excellent, very good, good	3,268 (91.9)	290 (8.2)	
Fair, poor	604 (82.7)	126 (17.3)	<0.0001
Annual household income			
$0–$25,000	837 (82.8)	174 (17.2)	
$25,000–$75,000	1,816 (90.7)	186 (9.3)	
$75,000+	1,219 (95.6)	56 (4.4)	<0.0001
Felt lonely			
Yes	494 (84.9)	88 (15.1)	
No	3,378 (91.2)	328 (8.9)	<0.0001

Values are presented as number (%) unless otherwise indicated.

**Cognitive status** (binary) was operationalized by using the variable Cogtot35, an HRS summary score of several cognitive tests (Ofstedal and Herzog 2005), dichotomized at 23, its observed mean. Less than 23 was considered low normal, mild, or worse loss of cognitive function.

**Alcohol consumption** was based on the question, “Do you ever drink any alcoholic beverages such as beer, wine, or liquor?” A binary variable was created reflecting responses (“yes”) versus (“no” or “never have used alcohol”).

**Self-rated health** was based on a single-item question and categorized as a binary variable with health reported as excellent, very good, or good versus fair or poor. It was included as an umbrella measure to capture perceived health status that encompasses many health conditions and dimensions.

**Loneliness** was based on a single-item question and categorized as a binary variable. It was included as an umbrella measure to capture perceived loneliness. The question asked, “Now think about the past week and the feelings you experienced. Please tell me if each of the following was true for you much of the time during the past week. Much of the time during the past week you felt lonely. Would you say yes, or no?”

**Annual household income**, an HRS composite, imputed measure (3 categories), was not included in the core 6 variables because education and income were highly related (*P* < 0.0001) but was a candidate predictor because the relationship between edentulism and low income or poverty is well established (NIH 2021).

### Statistical Analyses

**Figure 2.**
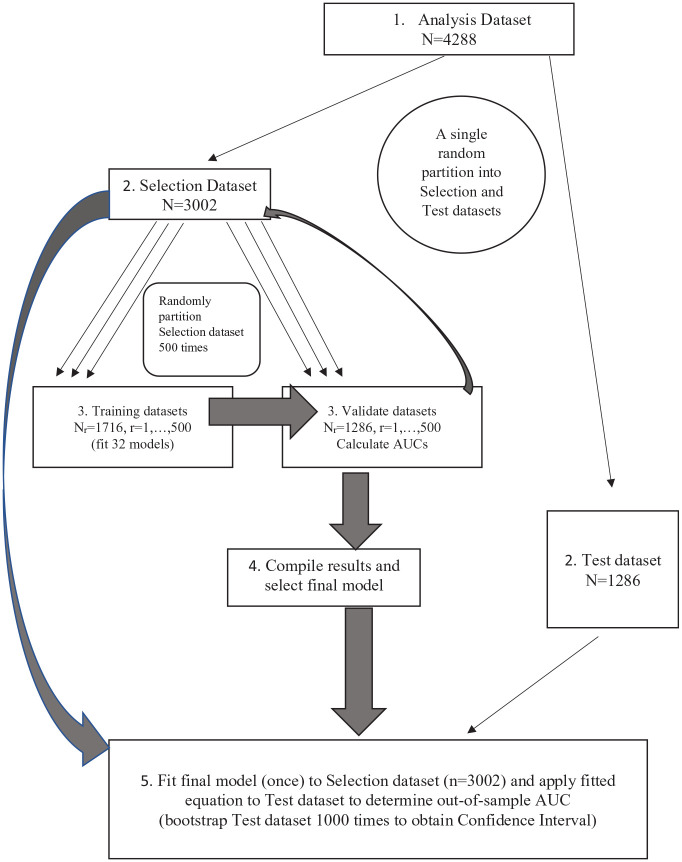
The process of model selection and assessment of generalized performance prediction of logistic regression model for 12-y tooth loss prediction. AUC, area under the curve.

Step 1 in the prediction modeling (Fig. 2) created the analytic data set with complete data (*n* = 4,288) that had an ID for linking across years, 12-y tooth loss (binary outcome), and 2 sets of explanatory variables. The first set consisted of our 6 established variables and the second set our 5 candidate predictor variables. Thus, we investigated 32 logistic regression models that also included all possible combinations of the 5 variables from the second set. In step 2, we randomly partitioned the analysis data set into 2 data sets: Selection (70%, i.e., *n* = 3,002) and Test (30%, i.e., *n* = 1,286). The Test data were not used for model selection; rather, they were only used to estimate the final prediction criterion, area under the curve (AUC) for the selected model.

In step 3, we applied Monte Carlo cross-validation (Xu and Goodacre 2018) to assess and compare the prediction performance of the 32 models. In this step, the Selection data set was randomly partitioned 500 times into Training (*n* = 1,716, 57%) and Validate (*n* = 1,286, 43%) data sets. In each replicate partition, the 32 logistic models were fitted to the Train data, and their fitted model equations were used to predict the Validate data using AUC to quantify model prediction performance.

In step 4, the prediction performance results were summarized to select the final model. We calculated the mean AUC (and 10th and 90th percentiles for reporting purposes) from the 500 Validate data sets for each model. The “best” model was the one with the largest mean AUC for Validate data across the 500 random partitions. If there were ties in AUC (to 3 decimal places) between 2 or more models having different number of predictors, then the more parsimonious model was considered superior as additional variables do not appreciably improve prediction.

Finally, in step 5, the generalized prediction performance of the selected “best” logistic regression model was determined using updated regression coefficients from its fit to the Selection data set (*n* = 3,002). The final, recalibrated AUC of the best model was obtained by applying the updated fitted model to the Test data (*n* = 1,286 from step 2). We calculated a bootstrap 95% confidence interval (CI) for the final AUC by applying the updated model fit to 1,000 with-replacement samples of size *n* = 1,286 from the test data. The mean of the 1,000 AUCs was taken as the final AUC, and the 2.5th and 97.5th percentiles provide the lower and upper 95% confidence bounds. An AUC above 0.70 was considered to give valid prediction.

## Results

Among the dentate 2006 participants, 438 died prior to 2018. Of the 6,576 dentate 2006 participants with known edentulous status in 2018, 2,288 were deleted because they had 1 or more missing covariates, with sample sizes as follows: cognition (*n* = 2150), loneliness (*n* = 165), income (*n* = 137), smoking (*n* = 57), alcohol (*n* = 17), dental utilization (*n* = 7), and self-rated general health (*n* = 3). Notably, smokers and individuals with annual income of $75,000+ were more substantially prevalent in the incomplete than in the analysis data set (Appendix Table 1). Among the 4,288 participants in the complete analysis data set, 123 were interviewed in a nursing home and 188 had a proxy answer questions.

The 12-y incidence of edentulism was 9.7%. At baseline, there was no significant difference between those who later became edentulous and those who remained dentate by gender and mean age. In 2006, the mean age (SD) for those who retained any teeth was 66.3 (8.4) y, and those who became edentulous, 66.7 (8.7) y. There were significant differences between these 2 groups for the other characteristics evaluated. A greater proportion of edentulous adults were African American or Hispanic compared to Caucasians, were more likely to have less than a college education and lower income, had a less favorable total cognition score, rated their health as fair or poor, and were less likely to have had regular dental care. They were more likely to report not drinking alcohol, being current smokers, and being lonely (Table 1).

**Figure 3.**
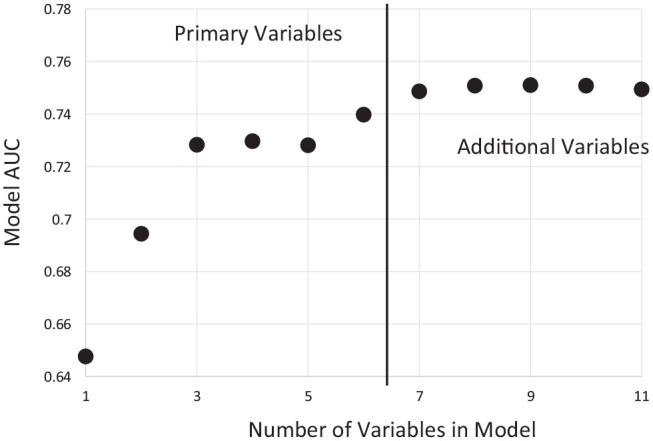
Plot of mean area under the curve (AUC) by number of variables in the model for each combination of variables. Best models from step 3 mean of 500 iterations are shown. Models to the right of the vertical bar all contain the original set of 6 variables.

In the Monte Carlo cross-validation, model selection process (steps 3 and 4), the 32 models applied to the 500 replicate training data sets (*n* = 1,716) included 1 model with only the original set of 6 variables and 31 models that included all possible combinations of the 5 candidate predictors for a total of 7 to 11 variables per model. In each random partition, the AUC was generated for each model to compare predictive ability. The mean AUC ranged from 0.739 to 0.751 for the 32 models (Appendix Table 2). The baseline, 6-variable model had mean AUC = 0.740 (Appendix Table 3), and the model chosen as the best one, which added cognition, had mean AUC = 0.749, a modest improvement. We selected the 7-variable model with cognition as the best model based on parsimony as there was negligible AUC improvement from adding more variables (Figure 3). Its updated model fit to the Selection data provided the final prediction equation, p = exp(*L*)/[1 + exp(*L*)], where p is the predicted probability of 12-y incident edentulism and

$$\begin{array}{l} L=-4.866+0.472R_{1}+0.322R_{2}\\ \;\;-0.130R_{3}+0.078G+0.158A\\ \;\;+\;0.908E_{0}+0.646E_{1}+1.261S_{0}\\ \;\;+0.389S_{1}+0.850D+0.599C \end{array}$$

with numbers being regression coefficients from step 5 and letters being predictor variables with notations in Table 2. Thus, an older adult’s probability of becoming edentulous in 12 y can be calculated by entering age in years divided by 10 for *A* and 0/1 indicators for all other characteristics; for example, risk levels of 67-y-olds are shown Appendix Table 4. Finally, the recalibrated AUC estimate was 0.748 (95% CI, 0.715–0.781). Defining the cutpoint probability of incident edentulism at 0.090, the observed value in the test data set, the estimated corrected sensitivity and specificity of the final model was 72.3% and 67.6%, respectively. Appendix Figure 1 depicts the receiver operating characteristic (ROC) curve. The positive predictive value was 19.9% and the negative predictive value was 95.7% (Appendix Table 5). In support of the split-sample approach, we note that, owing to random partitioning in step 2, the distribution of predictors and outcome in the Selection and Test data sets was similar (Appendix Table 6).

**Table 2.** Results of the 7-Variable Prediction Model for 12-y Edentulism among Older Adults in the Health and Retirement Study.

Variable	Type III *P* Value	Odds Ratio (Confidence Interval)
Race/ethnicity	0.045	
Caucasian		Reference
African American (*R*_1_)		1.60 (1.14–2.26)
Hispanic (*R*_2_)		1.38 (0.92–2.08)
Other (*R*_3_)		0.88 (0.36–2.15)
Gender	0.57	
Female		Reference
Male (*G*)		1.08 (0.83–1.41)
Age per 10 y (*A*)	0.06	1.17 (1.00–1.38)
Education/degree	0.001	
No high school degree (*E*_0_)		2.48 (1.52–4.06)
< College (*E*_1_)		1.91 (1.28–2.84)
College +		Reference
Smoking	<0.0001	
Current (*S*_0_)		3.53 (2.44–5.11)
Former (*S*_1_)		1.48 (1.10–1.97)
Never		Reference
Dental care	<0.0001	
Seen dentists irregularly (*D*)		2.34 (1.78–3.08)
Seen dentist within 2 y		Reference
Cognition	<0.0001	
Total cognition score ≥23		Reference
Total cognition score <23 (*C*, mild-severe loss)		1.82 (1.38–2.41)

In the selected, best model, being a nonsmoker and having regular dental care were both modifiable attributes that predicted lower odds of incident edentulism (Table 2). In terms of risk, current smokers had 3.53 (95% CI, 2.44–5.11) times the odds of being edentulous 12 y later than never smokers, and older adults who saw dentists irregularly had 2.34 (95% CI, 1.78–3.08) times the odds of incident edentulism than those who had seen dentists within the previous 2 y. Participants with mild to severe cognition loss had 1.82 (95% CI, 1.38–2.41) times the odds to become edentulous than those without cognitive decline defined as above. Among nonmodifiable demographic variables, gender and age were not statistically significantly associated with incident edentulism, whereas lower degree of education and African American race (vs. Caucasian) were significantly associated with increased incident edentulism.

## Discussion

Monte Carlo cross-validation with the use of external test data found that a 7-variable model including cognition and 2 modifiable risk factors, smoking and irregular dental care use, predicts 12-y edentulism among older adults in the United States with good accuracy. Demographic factors are generally used as covariates in building disease prediction models. Our finding that adding cognition to this set improves the prediction was noteworthy, even though the improvement in terms of classification probabilities was modest.

The risk prediction model could be used to estimate an individual’s probability or be used as a screening tool to predict 12-y incident edentulism versus remaining dentate based on their characteristics. Our results suggest the tool can rule out 12-y edentulism with over 95% accuracy while the prognosis of becoming edentulous is much less certain (positive predictive value of 20%). However, this level of risk communicated by a provider to a patient—“1 of 5 adults like you may lose all their teeth within 12 years”—may be sufficiently alarming to motivate the patient to stop smoking and/or see a dentist regularly to reduce their risk of becoming edentulous.

Cognitive function was included as a candidate predictor because of the increasing prevalence of dementia and other memory loss conditions among older adults. Potential biological mechanisms for a relationship between tooth loss and neurocognitive disorders have been examined (Harding and Singhrao 2022). Recent meta-analyses (Fang et al. 2018) and prospective studies suggest that oral diseases and tooth loss may increase risk of dementia (Han et al. 2020). Han et al. (2020) analyzed this relationship for edentulism in HRS data through 2014. A longitudinal cohort study among South Korean older adults reported an association of early stage cognitive impairment with increases in tooth loss and decreases with periodontal treatment (Yoo et al. 2019). Cerutti-Kopplin et al. (2016) in a systematic review (*n* = 10 studies) showed that adults with a suboptimal dentition (<20 teeth) had a higher risk for cognitive decline than those with >20 teeth. Chen et al. (2018) showed tooth loss conferred 1.34 times greater risk of developing dementia, with an increasing number of teeth lost increasing relative risk. Oh et al. (2018) suggest that having more teeth is associated with an almost 50% lower risk of dementia. However, the quality of the evidence was rated as very low. Recently, Qi and colleagues (2021) studied the longitudinal relationship between tooth loss and cognitive impairment. However, not all studies control for education, income, smoking, and self-reported health.

Self-rated health has been found to be a good prognostic measure for clinical outcomes (Fayers and Sprangers 2002). Muhammad and Srivastava (2022) found that edentulism was associated with poor self-rated health among older adults and, to a lesser extent, low psychological health and low subjective well-being. Medina-Solís et al. (2014) found edentulism was significantly associated with bad/very bad self-rated health.

Loneliness, 1 of 2 psychosocial factors included in the HRS Core, is a well-established risk factor for poor health outcomes (Ong et al. 2016). Prior longitudinal analysis in the United States has not accounted for psychosocial determinants of edentulism.

In this and other studies, alcohol consumption was found to be inversely associated with tooth loss (Heegaardet al. 2011) and edentulism (Weintraub et al. 2019) compared to abstainers. In another study, low alcohol consumption was associated with a higher risk of poor self-reported health. To explain this finding, the authors conjectured that non- and former drinkers might abstain due to illness (Medina-Solís 2014). In a different context, alcohol would be expected to be a risk factor for edentulism like smoking, because drinking and smoking often occur together. Alcohol consumption is a risk factor for oral cancer and periodontitis (NIH 2021).

This analysis has public health implications because of the aging population and the increase in proportion of the population with cognitive decline (Gerritsen et al. 2010; Kemle and Ackermann 2018). Even though the prevalence of edentulism has been declining in the United States (NIH 2021), it still affects over 6 million older adults (Dye et al. 2019). Because of the relationship of edentulism with lower education, level, race/ethnicity, smoking, and irregular dental utilization, factors associated with oral health disparities, these findings are not likely to change soon. In addition, older adults who have cognitive and functional limitations may have difficulty obtaining access to a traditional, fixed dental office and oral health professionals educated appropriately to care for people with special health care needs.

The strengths of this study included the ability to conduct a longitudinal analysis using prediction modeling methods with 12 y of follow-up, a large sample size, traditional demographic variables, and additional domains. The prediction modeling methods used tested the final model on a separate, independent data set. The prediction model can be used in clinical settings to predict 12-y tooth loss, given an individual’s characteristics, including cognition plus modifiable risk factors. Health care providers can also advise patients with fewer teeth to stop smoking and to see their dentist.

The availability of rich sociodemographic, health, behavioral, and social variables in the large ongoing HRS allowed examination of the impact of oral–systemic relationships over time. Such longitudinal studies can have profound implications for prevention. Conversely, a limitation of using secondary data was the constraints of available variables. Some variables are collected on a preestablished schedule and not available at every data collection cycle or as part of the HRS Core. The edentulism question is administered every 6 y, in every third 2-y data collection wave.

We did not test for interaction effects. For example, Russell et al. (2013) have developed a complex conceptual model to describe the relationship between sex, gender, and tooth loss. They discussed a potential gender effect with tooth loss over time because of differential exposure to behaviors such as tobacco and alcohol, social factors, dental care use, and oral hygiene. A review by Emami et al. (2013) identified socioeconomic factors and female gender contributing to the prevalence of complete tooth loss, along with age, education, access to dental care, dentist/population ratios, and insurance coverage. Roberto et al. (2019), in a systematic review, found men were at lower risk of edentulism and there was no significant difference in risk by race/ethnicity/skin color. In the United States, differences in edentulism by gender have attenuated overall but vary by age (NIH 2021).

There are limitations to this analysis of self-reported data. Clinical dental data were not available such as number of teeth present prior to becoming edentulous, oral hygiene, or periodontal status. Some additional HRS dental data were collected in 2008 and 2018 from supplemental questions administered to much smaller subsamples of the population. Importantly, we were not able to analyze any Asian or different Hispanic ethnic groups nor Native Americans, who have had the highest prevalence of edentulism (Wu et al. 2012). Nor did we have access to location of residence, which is important because edentulism does vary by state in the United States (NIH 2021), and rural versus nonrural residence (Saman et al. 2014). Similarly, government-sponsored dental Medicaid coverage for adults varies by state and over time, which affects access to care for low-income populations (Gerritsen et al. 2010). At age 65, adults in the United States become eligible for Medicare health coverage, but Medicare does not include routine dental care. Retirees usually lose any employer-based private dental care benefits. Finally, the analysis excluded a large number of HRS participants with missing cognitive status. People with dementia could be less likely to be in the data set, affecting results.

Other variables from the HRS Core data could have been considered. For example, diabetes and other systemic diseases are associated with periodontal disease and tooth loss (Patel et al. 2013). We separately evaluated the relationship between diabetes and self-rated health and found them to be highly associated. However, self-rated health is more holistic and captures more than diabetes status.

The analysis excluded adults under age 51 y. Therefore, caution should be used in applying our prediction model to adults of younger ages. The relative importance of risk factors for becoming edentulous could be different for people who become edentulous at younger ages. Adults who were still dentate in 2006 may have had more favorable life circumstances or behaviors than people who were already edentulous. With these limitations in mind, the large sample size and national sample should provide generalizability of the results for initially dentate adults, who were, on average, age 65 y at baseline and queried 12 y later. Future research could evaluate the prediction model in diverse samples, including populations at greater risk for oral diseases.

A previous study of factors associated with 6-y edentulism incidence using HRS data found that 5% of participants became edentulous between 2006 and 2012 (Weintraub et al. 2019). Findings for the common predictors used in this and the current study were similar.

To help people maintain natural teeth, clinicians should provide a prevention focus to their patients experiencing cognitive decline, especially if they are in other demographic risk categories. Education and collaboration with caregivers at home and the extended care interdisciplinary team may be necessary to ensure daily oral care and reduce risks from xerostomic medications, tobacco use, and cariogenic diet. Personal and professionally applied preventive oral care will need to be increased. Management will vary with the individuals’ dependency level and whether they are living at home or in a long-term care residence (Pretty et al. 2014).

## Conclusions

We used longitudinal, national HRS data and split-sample prediction modeling to identify the best set of variables to predict 12-y incidence of becoming edentulous among adults older than 50 y. The best prediction performance among 32 models with 6 to 11 candidate variables was determined using the AUC. In the 7-variable model, cognition information improved the 12-y prediction of becoming edentulous beyond the modifiable risk factors of smoking and dental care use, as well as nonmodifiable demographic factors of age, gender, race/ethnicity, and education.

## Author Contributions

J.S. Preisser, contributed to conception, design, data analysis and interpretation, drafted and critically revised the manuscript; K. Moss, contributed to conception, design, data acquisition, analysis, and interpretation, drafted and critically revised the manuscript; T.L. Finlayson, J.A. Jones, J.A. Weintraub, contributed to conception, design and data interpretation, drafted and critically revised the manuscript. All authors gave final approval and agree to be accountable for all aspects of the work.

## Supplemental Material

sj-docx-1-jct-10.1177_23800844221112062 – Supplemental material for Prediction Model Development and Validation of 12-Year Incident Edentulism of Older Adults in the United States Click here for additional data file. Supplemental material, sj-docx-1-jct-10.1177_23800844221112062 for Prediction Model Development and Validation of 12-Year Incident Edentulism of Older Adults in the United States by J.S. Preisser, K. Moss, T.L. Finlayson, J.A. Jones and J.A. Weintraub in JDR Clinical & Translational Research
