# Supplementary material for: Prediction Model Development and Validation of 12-Year Incident Edentulism of Older Adults in the United States
Source: JDR Clin Trans Res. 2022 Aug 9;8(4):384–93. doi: 10.1177/23800844221112062 (PMC10504805; doi:10.1177/23800844221112062)
Supplement: sj-docx-1-jct-10.1177_23800844221112062 – Supplemental material for Prediction Model Development and Validation of 12-Year Incident Edentulism of Older Adults in the United States [file sj-docx-1-jct-10.1177_23800844221112062.docx]

**APPENDIX**

**Prediction Model Development and Validation of 12-year Incident Edentulism**

**of Older Adults in U.S.**

J. S. Preisser^1^, K. Moss^2^, T. L. Finlayson^4^, J. A. Jones^4^, J. A. Weintraub^5^

^1^J.S. Preisser, Biostatistics, University of North Carolina Gillings School of Global Public Health, Chapel Hill, North Carolina, United States

^2^K. Moss, Division of Comprehensive Oral Health University of North Carolina at Chapel Hill, Adams School of Dentistry, Chapel Hill, North Carolina, United States
^3^T.L. Finlayson, Health Management and Policy, San Diego State University School of Public Health, San Diego, California, United States
^4^J.A. Jones, University of Detroit Mercy, Detroit, Michigan, United States

^5^J.A. Weintraub, Division of Pediatric and Public Health, University of North Carolina at Chapel Hill, Adams School of Dentistry, Chapel Hill, North Carolina, United States

Table A1. Frequency Distribution of Study Variables by Complete and Incomplete Data

|  | Complete Data  N=4288 | Incomplete  Data  N=2288 |
| --- | --- | --- |
| Race  Caucasian  African American  Hispanic  Other | 3198 (74.6%)  547 (12.8%)  441 (10.3%)  102 (2.4%) | 1671 (73.0%)  318 (13.9%)  231 (10.1%)  68 (3.0%) |
| Female  Male | 2491 (58.1%)  1797 (41.9%) | 1443 (63.1%)  845 (36.9%) |
| Mean(SD) Age, Years | 66.3 (8.4) | 61.2 (4.2) |
| Education/Degree  No High School Degree  < College  College + | 615 (14.3%)  2474 (57.7%)  1199 (28.0%) | 303 (13.2%)  1336 (58.4%)  649 (28.4%) |
| Smoking  Current  Former  Never | 398 (9.3%)  1796 (41.9%)  2094 (48.8%) | 297 (13.3%)  897 (40.2%)  1037 (46.5%) |
| Seen DDS within 2 Years  Seen DDS >2 Years | 3319 (77.4%)  969 (22.6%) | 1752 (76.8%)  529 (23.2%) |
| Ever Drink Alcohol (Yes)  No | 2490 (58.1%)  1798 (41.9%) | 1309 (57.6%)  962 (42.4%) |
| Total Cognition Score  < 23  23+ | 1424 (33.2%)  2864 (66.8%) | 65 (47.1%)  73 (52.9%) |
| Self-Rated Health  Excellent, Very Good, Good  Fair, Poor | 3558 (83.0%)  730 (17.0%) | 1878 (82.2%)  407 (17.8%) |
| Annual Household Income  $0-$25,000  $25,000-$75,000  $75,000+ | 1011 (23.6%)  2002 (46.7%)  1275 (29.7%) | 416 (19.3%)  892 (41.5%)  843 (39.2%) |
| Felt Lonely (Yes)  No | 582 (13.6%)  3706 (86.4%) | 275 (13.0%)  1848 (87.1%) |

Table A2. AUC for 32 candidate models from the model development and validation process with all 6 established variables included and combinations of the 5 candidate predictors.

| Model | N Variables in Model | Age, Race, Gender, Smoke, Education, Dental Visit | Alcohol | Self-rated Health | Income | Lonely | Cognition | Validate AUC | Validate AUC 10th Percentile | Validate AUC 90th Percentile |
| --- | --- | --- | --- | --- | --- | --- | --- | --- | --- | --- |
| 1 | 6 | X |  |  |  |  |  | 0.740 | 0.715 | 0.765 |
| 2 | 7 | X | X |  |  |  |  | 0.742 | 0.718 | 0.766 |
| 3 | 7 | X |  | X |  |  |  | 0.742 | 0.717 | 0.766 |
| 4 | 7 | X |  |  | X |  |  | 0.739 | 0.715 | 0.763 |
| 5 | 7 | X |  |  |  | X |  | 0.741 | 0.716 | 0.766 |
| 6 | 7 | X |  |  |  |  | X | 0.749 | 0.724 | 0.772 |
| 7 | 8 | X | X | X |  |  |  | 0.743 | 0.720 | 0.766 |
| 8 | 8 | X | X |  | X |  |  | 0.742 | 0.718 | 0.766 |
| 9 | 8 | X | X |  |  | X |  | 0.743 | 0.719 | 0.767 |
| 10 | 8 | X | X |  |  |  | X | 0.751 | 0.728 | 0.773 |
| 11 | 8 | X |  | X | X |  |  | 0.740 | 0.717 | 0.764 |
| 12 | 8 | X |  | X |  | X |  | 0.742 | 0.718 | 0.766 |
| 13 | 8 | X |  | X |  |  | X | 0.749 | 0.726 | 0.773 |
| 14 | 8 | X |  |  | X | X |  | 0.739 | 0.716 | 0.764 |
| 15 | 8 | X |  |  | X |  | X | 0.748 | 0.724 | 0.772 |
| 16 | 8 | X |  |  |  | X | X | 0.749 | 0.724 | 0.772 |
| 17 | 9 | X | X | X | X |  |  | 0.742 | 0.718 | 0.766 |
| 18 | 9 | X | X | X |  | X |  | 0.744 | 0.720 | 0.767 |
| 19 | 9 | X | X | X |  |  | X | 0.751 | 0.728 | 0.774 |
| 20 | 9 | X |  | X | X | X |  | 0.740 | 0.717 | 0.764 |
| 21 | 9 | X |  | X | X |  | X | 0.748 | 0.725 | 0.771 |
| 22 | 9 | X |  |  | X | X | X | 0.748 | 0.724 | 0.772 |
| 23 | 9 | X | X |  | X | X |  | 0.742 | 0.718 | 0.766 |
| 24 | 9 | X | X |  |  | X | X | 0.751 | 0.728 | 0.774 |
| 25 | 9 | X |  | X |  | X | X | 0.749 | 0.726 | 0.773 |
| 26 | 9 | X | X |  | X |  | X | 0.750 | 0.727 | 0.774 |
| 27 | 10 | X | X | X | X | X |  | 0.742 | 0.719 | 0.766 |
| 28 | 10 | X |  | X | X | X | X | 0.748 | 0.725 | 0.771 |
| 29 | 10 | X | X |  | X | X | X | 0.750 | 0.727 | 0.775 |
| 30 | 10 | X | X | X |  | X | X | 0.751 | 0.728 | 0.774 |
| 31 | 10 | X | X | X | X |  | X | 0.750 | 0.728 | 0.773 |
| 32 | 11 | X | X | X | X | X | X | 0.749 | 0.727 | 0.773 |

Table A3: Mean (80% CI)* AUC for Best 1-6 baseline variable models.

| Model | Variable(s) | Mean  AUC | 10^th^ Percentile  AUC | 90^th^ Percentile  AUC |
| --- | --- | --- | --- | --- |
| 1 variable | Education | 0.648 | 0.625 | 0.670 |
| 2 variable | Education, Smoking | 0.694 | 0.668 | 0.720 |
| 3 variable | Education, Smoking, Dental Utilization | 0.728 | 0.704 | 0.754 |
| 4 variable | Education, Smoking, Dental Utilization, Sex | 0.727 | 0.703 | 0.750 |
| 5 variable | Education, Smoking, Dental Utilization, Sex, Age | 0.728 | 0.703 | 0.753 |
| 6 variable | Education, Smoking, Dental Utilization, Sex, Age, Race | 0.740 | 0.715 | 0.765 |

*500 Iterations

Figure A1. ROC curve with 80% prediction bands based on 10^th^ and 90^th^ bootstrap percentiles for the prediction of incident 12-year edentulism among older adults in the Health and Retirement Study, 2006-2018, based on model development and validation and, using independent test data, recalibration.


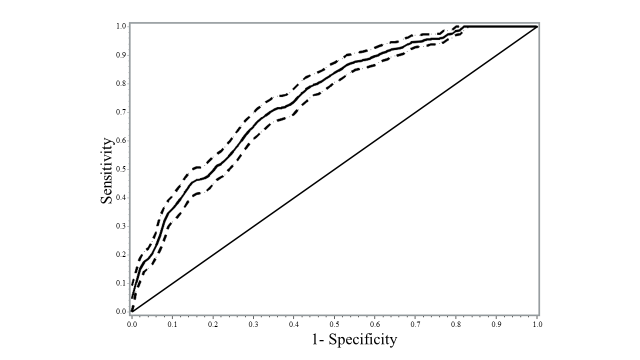


Table A4. The number of adults aged 67 out of 1000^1^ predicted to have edentulism in 12 years based on the 7-variable model

|  | | | | Current Smoker | | Former Smoker | | Never Smoker | |
| --- | --- | --- | --- | --- | --- | --- | --- | --- | --- |
|  | | Dental care | | regular | irregular | regular | irregular | regular | irregular |
| Race | Gen | Educ | Cog |  |  |  |  |  |  |
| Cauc | F | N | L | 261 | 453 | 129 | 257 | 91 | 190 |
|  |  |  | H | 163 | 312 | 75 | 160 | 52 | 114 |
|  |  | S | L | 214 | 389 | 102 | 210 | 72 | 153 |
|  |  |  | H | 130 | 259 | 59 | 128 | 41 | 90 |
|  |  | D | L | 125 | 250 | 56 | 122 | 39 | 86 |
|  |  |  | H | 73 | 155 | 32 | 71 | 22 | 49 |
|  | M | N | L | 277 | 472 | 138 | 272 | 98 | 202 |
|  |  |  | H | 174 | 329 | 81 | 170 | 56 | 122 |
|  |  | S | L | 227 | 408 | 110 | 224 | 77 | 163 |
|  |  |  | H | 139 | 274 | 63 | 137 | 44 | 97 |
|  |  | D | L | 134 | 265 | 61 | 131 | 42 | 93 |
|  |  |  | H | 78 | 165 | 34 | 77 | 23 | 53 |
| AA | F | N | L | 362 | 570 | 192 | 357 | 138 | 273 |
|  |  |  | H | 237 | 421 | 115 | 234 | 81 | 171 |
|  |  | S | L | 304 | 505 | 154 | 299 | 110 | 224 |
|  |  |  | H | 193 | 359 | 91 | 190 | 64 | 137 |
|  |  | D | L | 186 | 348 | 87 | 183 | 61 | 132 |
|  |  |  | H | 112 | 227 | 50 | 109 | 34 | 77 |
|  | M | N | L | 380 | 589 | 204 | 375 | 148 | 289 |
|  |  |  | H | 252 | 440 | 123 | 248 | 87 | 182 |
|  |  | S | L | 320 | 524 | 165 | 316 | 118 | 238 |
|  |  |  | H | 206 | 377 | 98 | 202 | 68 | 147 |
|  |  | D | L | 198 | 366 | 94 | 195 | 65 | 141 |
|  |  |  | H | 119 | 241 | 54 | 117 | 37 | 83 |
| Hisp | F | N | L | 328 | 533 | 170 | 323 | 122 | 245 |
|  |  |  | H | 211 | 385 | 101 | 208 | 71 | 151 |
|  |  | S | L | 273 | 468 | 136 | 269 | 96 | 199 |
|  |  |  | H | 171 | 326 | 79 | 168 | 55 | 120 |
|  |  | D | L | 164 | 315 | 76 | 162 | 53 | 115 |
|  |  |  | H | 98 | 202 | 43 | 96 | 30 | 67 |
|  | M | N | L | 345 | 552 | 181 | 341 | 130 | 259 |
|  |  |  | H | 225 | 404 | 108 | 221 | 76 | 161 |
|  |  | S | L | 289 | 487 | 145 | 284 | 103 | 212 |
|  |  |  | H | 182 | 343 | 85 | 179 | 59 | 129 |
|  |  | D | L | 175 | 332 | 82 | 172 | 57 | 124 |
|  |  |  | H | 105 | 215 | 47 | 103 | 32 | 72 |
| Other | F | N | L | 237 | 421 | 115 | 233 | 81 | 171 |
|  |  |  | H | 146 | 285 | 67 | 143 | 46 | 102 |
|  |  | S | L | 193 | 359 | 91 | 190 | 63 | 137 |
|  |  |  | H | 116 | 235 | 52 | 114 | 36 | 80 |
|  |  | D | L | 111 | 227 | 50 | 109 | 34 | 77 |
|  |  |  | H | 64 | 139 | 28 | 63 | 19 | 44 |
|  | M | N | L | 251 | 440 | 123 | 247 | 87 | 182 |
|  |  |  | H | 156 | 301 | 72 | 153 | 50 | 109 |
|  |  | S | L | 205 | 377 | 98 | 202 | 68 | 146 |
|  |  |  | H | 124 | 249 | 56 | 122 | 39 | 86 |
|  |  | D | L | 119 | 241 | 54 | 117 | 37 | 82 |
|  |  |  | H | 69 | 148 | 30 | 68 | 21 | 47 |

^1^The numbers in the table are determined by multiplying the model predicted probability of 12-year edentulism by 1000. Race=Race/Ethnicity (Cauc=Caucasian, AA=African American, Hisp=Hispanic, Other); Gen=Gender (F=Female, M=Male); Educ=Education (N = No High School Degree, S = High School degree or some college (*E_1_*), D=Four year college or more); Cog=Cognition (L=Total Cognition Score < 23, H=Total Cognition Score > 23); Smok=Smoking (current; former; never); DC=Dental Care (regular=Seen dentist within 2 Years, Irregular= Seen dentists irregularly).

Table A5. Classification probabilities for prediction of 12-year edentulism among older adults in HRS

|  | **Prob** | **Correct^1^** | | **Incorrect** | | **Percentages** | | | |
| --- | --- | --- | --- | --- | --- | --- | --- | --- | --- |
|  | **Level** | **Event^2^** | **Non-** | **Event^2^** | **Non-Event** | **Sensi-** | **Speci-** | **Pos** | **Neg** |
|  |  |  | **Event** |  |  | **tivity** | **ficity** | **Pred** | **Pred** |
| Without Cognition^3^ | **0.09** | 207 | 1873 | 93 | 829 | 69 | 69.3 | 20.0 | 95.3 |
| With Cognition^4^ | **0.09** | 217 | 1827 | 83 | 875 | 72.3 | 67.6 | 19.9 | 95.7 |

**^1^Correct** = Correct prognosis**; ^2^Event** = 12 year incident edentulism; ^3^Base 6-variable model; ^4^Seven-variable model that includes Cognition

**Details of Calculation of Positive and Negative Predictive Values**

| **6-var model, without Cognition** | Prognosis is edentulism | Prognosis is dentate | Total |
| --- | --- | --- | --- |
| True edentulism | 207 | 93 | 300 |
| True dentate | 829 | 1873 | 2702 |
| Total | 1036 | 1966 | 3002 |

Pos Pred = P(truly edent|prognosis of edent) = 207/1036 = .1998 -> 20.0%

Neg Pred =P(truly dentate|prognosis is dentate) = 1873/1966 = 0.953 -> 95.3%

| **7-var model, without Cognition** | Prognosis is edentulism | Prognosis is dentate | Total |
| --- | --- | --- | --- |
| True edentulism | 217 | 83 | 300 |
| True dentate | 875 | 1827 | 2702 |
| Total | 1092 | 1910 | 3002 |

Pos Pred = P(truly edent|prognosis of edent) = 217/1092 = .1987 -> 19.9%

Neg Pred =P(truly dentate|prognosis is dentate) = 1827/1910 = 0.9565 -> 95.7%

Table A6. Frequency Distribution of 2006 Characteristics of Participants by Dentate Status in 2018 stratified by Selection and Test datasets

|  | Selection Dataset (n=3002) | | Test Dataset (n=1286) | |
| --- | --- | --- | --- | --- |
| Characteristic in 2006 | Dentate in 2018 | Became Edentulous between  2006-2018 | Dentate in 2018 | Became Edentulous between  2006-2018 |
| Total | 2702 (90.0%) | 300 (10.0%) | 1170 (91.0%) | 116 (9.0%) |
| Race/Ethnicity  Caucasian  African American  Hispanic  Other | 2083 (92.6%)  311 (81.0%)  242 (9.0%)  66 (91.7%) | 166 (7.4%)  73 (19.0%)  55 (18.5%)  6 (8.3%) | 881 (92.8%)  143 (87.7%)  121 (84.0%)  25 (83.3%) | 68 (7.2%)  20 (12.3%)  23 (16.0%)  5 (16.7%) |
| Gender  Female  Male | 1581 (90.3%)  1121 (89.5%) | 169 (9.7%)  131 (10.5%) | 684 (92.3%)  486 (89.2%) | 57 (7.7%)  59 (10.8%) |
| Mean (SD) Age, Years (in 2006) | 66.2 (8.5) | 66.5 (8.6) | 66.4 (8.3) | 67.2 (8.9) |
| Education/Degree  No High School Degree  < College  College + | 325 (78.1%)  1569 (89.9%)  808 (96.1%) | 91 (21.9%)  176 (10.1%)  33 (3.9%) | 166 (83.4%)  653 (89.6%)  351 (98.0%) | 33 (16.6%)  76 (10.4%)  7 (2.0%) |
| Smoking  Current  Former  Never | 211 (75.1%)  1144 (90.1%)  1347 (92.8%) | 70 (24.9%)  126 (9.9%)  104 (7.2%) | 88 (75.2%)  472 (89.7%)  610 (94.9%) | 29 (24.8%)  54 (10.3%)  33 (5.1%) |
| Last Dentist Visit  Within 2 Years  >2 Years | 2176 (93.4%)  526 (78.4%) | 155 (6.7%)  145 (21.6%) | 920 (93.1%)  250 (83.9%) | 68 (6.9%)  48 (16.1%) |
| Ever Drink Alcohol  Yes  No | 1622 (92.4%)  1080 (86.6%) | 133 (7.6%)  167 (13.4%) | 680 (92.5%)  490 (88.9%) | 55 (7.5%)  61 (11.1%) |
| Total Cognition Score  < 23  23+ | 834 (82.8%)  1868 (93.6%) | 173 (17.2%)  127 (6.4%) | 354 (84.9%)  816 (93.9%) | 63 (15.1%)  53 (6.1%) |
| Self-rated Health  Excellent, Very Good, Good  Fair, Poor | 2288 (91.7%)  414 (81.7%) | 207 (8.3%)  93 (18.3%) | 980 (92.2%)  190 (85.2%) | 83 (7.8%)  33 (14.8%) |
| Annual Household Income  $0-$25,000  $25,000-$75,000  $75,000+ | 597 (82.9%)  1226 (90.0%)  879 (95.5%) | 123 (17.1%)  136 (10.0%)  41 (4.5%) | 240 (82.5%)  590 (92.2%)  340 (95.8%) | 51 (17.5%)  50 (7.8%)  15 (4.2%) |
| Felt Lonely  Yes  No | 341 (84.2%)  2361 (90.9%) | 64 (15.8%)  236 (9.1%) | 153 (86.4%)  1017 (91.7%) | 24 (13.6%)  92 (8.3%) |

*All unadjusted associations of characteristic with becoming edentulous have p-value<0.05 except for gender and age.
